# Supplementary material for: Multifunctional human visual pathway-replicated hardware based on 2D materials
Source: Nat Commun. 2024 Oct 5;15:8650. doi: 10.1038/s41467-024-52982-3 (PMC11455896; doi:10.1038/s41467-024-52982-3)
Supplement: Supplementary file 1 — Supplementary Information [file 41467_2024_52982_MOESM1_ESM.pdf]

Supplementary Information for

Multifunctional human visual pathway-replicated hardware based on 2D materials

Zhuiri Peng[1], Lei Tong[2], Wenhao Shi[1], Langlang Xu[1], Xinyu Huang[1], Zheng Li[1], Xiangxiang Yu[1], Xiaohan Meng[1], Xiao He[1], Shengjie Lv[1], Gaochen Yang[1], Hao Hao[3], Tian Jiang[3,*], Xiangshui Miao[1,4,*], Lei Ye[1,4,*]

[1]*School of Integrated Circuits, Wuhan National Laboratory for Optoelectronics, Huazhong University of Science and Technology, Wuhan, China*

[2]*Department of Electronic Engineering, Materials Science and Technology Research Center, The Chinese University of Hong Kong, Hong Kong, China*

[3]*College of Advanced Interdisciplinary Studies, National University of Defense Technology, Changsha, China*

[4]*Hubei Yangtze Memory Laboratories, Wuhan, China*

*Corresponding authors. Email: tjiang@nudt.edu.cn (T. J.); miaoxs@hust.edu.cn (X. M.); leiye@hust.edu.cn (L. Y.)

Supplementary Notes

Supplementary Note 1 | Characteristic statistics and analysis of unit devices in the array

1.1 Measurement setup

The device has four signal ports: drain (V_D), source (Ground), and split floating gate (V_{G1} and V_{G2}). V_D, V_{G1}, and V_{G2} are variables, and drain current (I_D) is the output. The light input is controlled by a monochromator and attenuator, and the incident wavelength (λ) and light intensity (P_{in}) can be adjusted.

When measuring the electrical characteristics under the dark state, different characteristics can be tested according to the different controlled inputs: (1) when $V_{G1} = V_{G2} = V_G$ and V_D is fixed, I_D–V_G transfer curves are tested (Figs. S2b, c, S6a, b); (2) fix V_G to test I_D–V_D output curves (Fig. S7); (3) apply V_G pulse to test I_D-number of V_G pulse (Figs. 1g, S6e, f, S8). (4) When $V_{G1} = -V_{G2}$, V_G pulse is applied to build p–n configuration and record I_D–V_D (Fig. S3b). (5) Fix V_D to test I_D–V_{G1}&V_{G2} mapping (equivalent gate voltage) (Fig. S3c).

The optoelectronic characteristics of devices can be measured by adjusting the wavelength and intensity of the light: (6) fix wavelength and equivalent gate voltage, change the light intensity and test I_D–V_D output curves (Figs. S4b, d–f, S10); (7) fix wavelength and V_D = 0 V, change the light intensity to measure I_{SC}–V_G, the short-circuit current with the change of equivalent gate voltage (Figs. S4g–i); (8) fix wavelength and V_D = 0 V, change equivalent gate voltage V_G to test I_{SC}–P_{in}, the short-circuit current with the light intensity (Figs. 1f, S5a, b); (9) fix wavelength and the light intensity, V_D = 0 V, to measure I_{SC}–V_{G1}&V_{G2}, the relationship between the short-circuit current and the independent equivalent double gate voltages (Figs. 1e, S5c, d, S11).

1.2 Characteristic statistics and analysis

1) Extraction of optical responsivity

The optical responsivity R is calculated from the formula, $R=\frac{I_{ph}}{P}$, where the net photocurrent $I_{ph} = I_{light} - I_{dark}$ (I_{light}, drain current under illumination. I_{dark}, drain current under the dark state), and the effective incident light power $P = P_{in} \cdot S_{active}$ (P_{in},

incident light power density. S_{active}, effective photosensitive area of the device about 100 μm^2). Since the short-circuit current I_{SC} of the device is 0 A under the dark state, I_{SC} under light is its net photocurrent. Fig. S4c extracts R–V_G, the relationship between the optical responsivity and the equivalent gate voltage.

2) Extraction of the memory window for floating gate transistor

The transfer curve has obvious hysteresis when a double sweeping gate voltage is tested, from which the memory window is obtained according to the voltage span under a certain drain current I_D. For example, the memory window extracted at 1 nA I_D is defined as memory window(V) @ 1 nA (Fig. S6c top). The memory window is about 7.3–10.3 V at I_D = 1 nA, showing remarkable storage capacity.

3) Extraction of ON/OFF ratio for transistor

When $V_{G1} = V_{G2} = V_G$, the maximum I_{Dmax} and minimum values I_{Dmin} of I_D for the transfer curve (I_D–V_G) represent the ON and OFF states of the transistor, respectively. The ON/OFF ratio of conductance (or drain current), $\frac{I_{Dmax}}{I_{Dmin}}$, reflects its ability to switch (Fig. S6c bottom). The transistor exhibits excellent gate control with an ON/OFF ratio of 10^8–10^9.

4) Calculation method of subthreshold swing for transistor

The subthreshold swing (*SS*) is a performance measure of the transition rate between the ON and OFF states of a transistor, representing the amount of change in gate voltage required for a ten-fold change in drain current. The *SS* is calculated in the linear region of transfer curves, and the calculation formula is: $SS = \frac{dV_G}{d\log I_D}$ .We tested and counted the *SS* that concentrates at 150 mV/dec for 100 units (Figs. S2b, S6d top).

5) Calculation method of carrier mobility

Carrier mobility refers to the average drift velocity of carriers per unit of electric field strength, which represents its conductive ability. In general, higher mobility results in faster working speed and higher cut-off frequency. Carrier mobility is inversely proportional to the effective mass of the carriers and the scattering probability. For the channel material in a transistor, carrier mobility can be extracted directly from transfer

curves. It is calculated as follows: $\mu_{\mathrm{gm}} = \frac{g_{\mathrm{m,max}} L}{W C_{\mathrm{G}} V_{\mathrm{D}}}$, where $g_{\mathrm{m,max}}$ is the maximum value of transconductance g_{m}, $g_{\mathrm{m}} = \frac{\mathrm{d}I_{\mathrm{D}}}{\mathrm{d}V_{\mathrm{G}}}$; W, L, the width (8 μm) and length (4 μm) of the channel; C_{G}, the gate capacitance per unit area, $C_{\mathrm{G}} = \frac{\varepsilon}{d}$; ε, d, the dielectric constant ($\varepsilon = \varepsilon_0 \cdot \varepsilon_{\mathrm{Al_2O_3}}$; vacuum dielectric constant, $\varepsilon_0 = 8.854187817\times10^{-12}$ F/m; relative permittivity of Al_2O_3, $\varepsilon_{\mathrm{Al_2O_3}} = 8.9$), and thickness (30 nm) of the gate dielectric. Carrier mobility of 100 units was measured and distributed between 35–42 $\mathrm{cm^2V^{-1}s^{-1}}$ (Fig. S6d bottom), verifying great performance uniformity of WSe_2 transistor.

6) Extraction of ON/OFF ratio for p–n junction

When $V_{\mathrm{G1}} = -V_{\mathrm{G2}} = V_{\mathrm{G}}$, the maximum I_{Dmax} and minimum values I_{Dmin} of I_{D} for the output curve (I_{D}–V_{D}) represents the forward conduction I_{Don} and reverse cut-off states $I_{\mathrm{Dcut\text{-}off}}$ of the p–n junction, respectively. The ON/OFF ratio of the p–n junction, $\frac{I_{\mathrm{Dmax}}}{I_{\mathrm{Dmin}}}$, reflects its rectification ability (Fig. S9c, top).

7) Estimation of programming energy for floating gate

During the programming process, the drain and source are grounded. The energy consumption only originates from the voltage pulse supplied to the gate. The voltage pulse is fixed at 1 V and 10 ms, with a gate leakage current of less than 0.1 nA. Therefore, the programming energy is estimated to be no more than 1 pJ/spike, calculated by $E_{\mathrm{program}} = V_{\mathrm{pulse}} \cdot I_{\mathrm{pulse}} \cdot t_{\mathrm{pulse}}$. The programming energy of the sparse neural network and the fully connected neural network (Fig. 3g) is estimated by performing 32 spikes on average per device for 64-level weights. Therefore, the programming energy per device is evaluated to 32 pJ. It consumes about nearly 0.9 nJ with 28 devices for sparse NN, while 2.3 nJ with 72 devices for full-NN.

8) Calculation for device usage

Device usage depends on the numbers of weights in the NN. For this 5× 8 × 4 NN, full connected one has 5× 8 + 8 × 4 = 72 weights, sparse connected one has only 28 weights (Fig. 3c, Source Data 3e).

Supplementary Note 2 | Principle and peripheral circuit design of Color processing hardware

2.1 Principle

According to the spectral sensitivity, cone cells can be divided into S, M, and L cone cells, which have peak response to blue (B), green (G), and red (R) light, respectively. Color information is encoded in a trichromatic manner through L, M, and S cone cells in the retina when first entering the eye, and then propagates in a color-opponency manner which is processed and analyzed by SO/DO CSRF in the retinal ganglion layer, LGN, V1, V4 and IT[1–3].

Retinal cone cells: convert the intensity of red, green, and blue light inputs into voltage signals — $\mathbf{R}(x, y)$, $\mathbf{G}(x, y)$, $\mathbf{B}(x, y)$.

Retinal ganglion cells: Adjacent unit signals from horizontal cells, bipolar cells, and amacrine cells are superimposed to obtain voltage signals of yellow (Y) and black (K). It is calculated as follows:

$$\mathbf{Y}(x, y) = \mathbf{R}(x, y) + \mathbf{G}(x, y)$$

$$\mathbf{K}(x, y) = \mathbf{R}(x, y) + \mathbf{G}(x, y) + \mathbf{B}(x, y)$$

Depending on potentiation or depression, there are signals such as $\mathbf{R}_{\pm}(x, y)$, $\mathbf{G}_{\pm}(x, y)$, $\mathbf{B}_{\pm}(x, y)$, $\mathbf{Y}_{\pm}(x, y)$, $\mathbf{K}_{\pm}(x, y)$.

Lateral geniculate nucleus (LGN): The color information is treated in opponency of red–green ($\mathbf{O}_{\mathrm{RG}}$ or $\mathbf{O}_{\mathrm{GR}}$), yellow–blue ($\mathbf{O}_{\mathrm{YB}}$ or $\mathbf{O}_{\mathrm{BY}}$), black and white ($\mathbf{O}_{\mathrm{K+}}$ or $\mathbf{O}_{\mathrm{K-}}$).

$$\begin{bmatrix}\mathbf{O}_{\mathrm{RG}}\\ \mathbf{O}_{\mathrm{YB}}\\ \mathbf{O}_{\mathrm{K+}}\end{bmatrix} = \begin{bmatrix}\frac{1}{\sqrt{2}} & -\frac{1}{\sqrt{2}} & 0\\ \frac{1}{\sqrt{6}} & \frac{1}{\sqrt{6}} & -\frac{2}{\sqrt{6}}\\ \frac{1}{\sqrt{3}} & \frac{1}{\sqrt{3}} & \frac{1}{\sqrt{3}}\end{bmatrix}\begin{bmatrix}\mathbf{R}_{+}\\ \mathbf{G}_{+}\\ \mathbf{B}_{+}\end{bmatrix} \quad (1)$$

$$\begin{bmatrix}\mathbf{O}_{\mathrm{GR}}\\ \mathbf{O}_{\mathrm{BY}}\\ \mathbf{O}_{\mathrm{K-}}\end{bmatrix} = \begin{bmatrix}\frac{1}{\sqrt{2}} & -\frac{1}{\sqrt{2}} & 0\\ \frac{1}{\sqrt{6}} & \frac{1}{\sqrt{6}} & -\frac{2}{\sqrt{6}}\\ \frac{1}{\sqrt{3}} & \frac{1}{\sqrt{3}} & \frac{1}{\sqrt{3}}\end{bmatrix}\begin{bmatrix}\mathbf{R}_{-}\\ \mathbf{G}_{-}\\ \mathbf{B}_{-}\end{bmatrix} \quad (2)$$

The receptive field of a SO cell can be described as a 2D Gaussian function:

$$\mathbf{RF}(x, y; \sigma) = \frac{1}{2\pi\sigma^2}\exp(-\frac{x^2+y^2}{2\sigma^2}) \quad (3)$$

The standard deviation σ controls the size of the receptive field.

The σ = 0.08 and σ = 0.12 is respectively added to responsivity distribution of small (3×3) and large (5×5) CSRF. Specifically, the small one has two values (0.04 and 0.008 A/W) and the large one has three values (0.007, 0.003 and 0.0001 A/W), constructing convolution kernels as below:

$$\begin{bmatrix} 0.008 & 0.008 & 0.008 \\ 0.008 & 0.04 & 0.008 \\ 0.008 & 0.008 & 0.008 \end{bmatrix}$$

$$\begin{bmatrix} 0.0001 & 0.0001 & 0.0001 & 0.0001 & 0.0001 \\ 0.0001 & 0.003 & 0.003 & 0.003 & 0.0001 \\ 0.0001 & 0.003 & 0.007 & 0.003 & 0.0001 \\ 0.0001 & 0.003 & 0.003 & 0.003 & 0.0001 \\ 0.0001 & 0.0001 & 0.0001 & 0.0001 & 0.0001 \end{bmatrix}$$

In each site, the responsivity value for R/G/B in the 3×3 or 5×5 CSRF is the same. The sign "+" / "–" in "R+, R–, G+, G–, B+, B–" represents potentiated/ depressed information at the corresponding site.

The signal of an opponency cell (SO) is the convolution of the opponency signal and its receptive field:

$$\mathbf{SO}(x, y) = \mathbf{O}(x, y) * \mathbf{RF}(x, y, \sigma) \tag{4}$$

Thus, the responses of each SO cell are expressed separately as:

$$\mathbf{SO}_{R+G-}(x, y) = \mathbf{O}_{RG}(x, y) * \mathbf{RF}(x, y, \sigma_{small})$$

$$\mathbf{SO}_{G+R-}(x, y) = \mathbf{O}_{GR}(x, y) * \mathbf{RF}(x, y, \sigma_{large})$$

$$\mathbf{SO}_{Y+B-}(x, y) = \mathbf{O}_{YB}(x, y) * \mathbf{RF}(x, y, \sigma_{small})$$

$$\mathbf{SO}_{B+Y-}(x, y) = \mathbf{O}_{BY}(x, y) * \mathbf{RF}(x, y, \sigma_{large})$$

$$\mathbf{SO}_{Bri.}(x, y) = \mathbf{O}_{K+}(x, y) * \mathbf{RF}(x, y, \sigma_{small})$$

$$\mathbf{SO}_{Dar.}(x, y) = \mathbf{O}_{K-}(x, y) * \mathbf{RF}(x, y, \sigma_{large})$$

σ_{small}, σ_{large}, small and large receptive fields.

In fact, the SO signal is get by adding the "R+G–" and "G–R+", which is equal to add full "R+" and full "G–". Therefore, the convolution kernels for "R+" and "G–" are respectively set to all positive and negative values, limited by single-color input of the testing facility.

Primary visual cortex (V1): The DO cells widely present here superpose the SO signals of the large and small receptive fields incoming from the LGN to obtain the DO signal.

$$\begin{bmatrix} \mathbf{DO}_{\text{R\&G}} \\ \mathbf{DO}_{\text{B\&Y}} \\ \mathbf{DO}_{\text{Gray}} \end{bmatrix} = \begin{bmatrix} rg & rg\cdot k & 0 & 0 & 0 & 0 \\ 0 & 0 & yb & yb\cdot k & 0 & 0 \\ 0 & 0 & 0 & 0 & g & g\cdot k \end{bmatrix} \begin{bmatrix} \mathbf{SO}_{\text{R+G-}} \\ \mathbf{SO}_{\text{G+R-}} \\ \mathbf{SO}_{\text{Y+B-}} \\ \mathbf{SO}_{\text{B+Y-}} \\ \mathbf{SO}_{\text{Bri.}} \\ \mathbf{SO}_{\text{Dar.}} \end{bmatrix} \tag{5}$$

k = 0.9, represents the difference in intensity between the large and small receptive fields. Coefficient rg, yb, g is the proportion of red–green, yellow–blue, and black–white SO signals transmitted from LGN to V1, respectively, reflecting the working state of the color processing visual pathway. If their values are very different from each other, color vision impairment will occur. For example, when $rg = 0.1$, $yb = 1$, and $g = 1$, the failure of the red–green information processing pathway will lead to the formation of red-green colorblindness (Fig. 2f).

The advanced visual cortex V2/V4/IT transforms the visual information encoded by DO CSRF into a trichromatic RGB space, and the color is extracted statistically by the color constant cells that collect the information of the large receptive field. The formula is an inverse transformation:

$$\begin{bmatrix} \mathbf{R} \\ \mathbf{G} \\ \mathbf{B} \end{bmatrix} = \begin{bmatrix} \frac{1}{\sqrt{2}} & -\frac{1}{\sqrt{2}} & 0 \\ \frac{1}{\sqrt{6}} & \frac{1}{\sqrt{6}} & -\frac{2}{\sqrt{6}} \\ \frac{1}{\sqrt{3}} & \frac{1}{\sqrt{3}} & \frac{1}{\sqrt{3}} \end{bmatrix}^{-1} \begin{bmatrix} \mathbf{DO}_{\text{R\&G}} \\ \mathbf{DO}_{\text{B\&Y}} \\ \mathbf{DO}_{\text{Gray}} \end{bmatrix} \tag{6}$$

At this time, the trichromatic proportion of HVS is the same as the RGB components. The color constancy information can be obtained by normalizing and pooling the whole through global statistics (simulation results).

2.2 Circuit design

According to the principle, we designed the color processing hardware and fabricated a PCBA. The hardware is divided into two modules: the retina-sensing module and the cortex-processing module.

The retina-sensing module (macroscopic image in Fig. S14) is composed of a 3 ×

3 or 5 × 5 SFG array (only part of 10 × 10 SFG array is under work) and a trans-impedance amplifier (TIA) to perform the photoelectric measurement based on photovoltaic effect, and current-voltage conversion and voltage output, respectively. Each column of drains is connected to the Ground, all sources is connected to the TIA to form a voltage signal output, and the double gate of each row is independently controlled by an external power supply to modify the optical responsivity of each unit. The cortex-processing module (macroscopic image in Fig. S12) is composed of a 10 × ten SFG array and ten TIAs, which perform the matrix multiplication operation of the front-end voltage input. Each column of drains is connected to form ten channels of voltage inputs, and each row of sources is connected to the TIA to form ten channels of voltage output, and the double gate of each row is independently controlled by an external power supply to adjust the conductivity of each unit. The peripheral processing module can perform the $m \times n$ matrix multiplication using only m columns and n rows.

The color processing hardware (supplementary Fig. S12) requires the use of one retina-sensing module (reused twelve times for positive/negative R/G/B under 3 × 3 and 5 × 5) and four cortex-processing modules (three for 6 × 3, one for 3 × 3). Among them, six 3 × 3 and 5 × 5 retina-sensing modules carry out photoelectric conversion of small and large receptive fields $\mathbf{R}_+$, $\mathbf{R}_-$, $\mathbf{G}_+$, $\mathbf{G}_-$, $\mathbf{B}_+$, $\mathbf{B}_-$, respectively. Limited by single-color input of the testing facility, the retina-sensing module is reused to record twelve voltage signals which are imported into subsequent circuits through a signal generator in experiment. The voltage outputs are connected to a 6 × 3 cortex-processing module for matrix multiplication (combined with equations 1–4) to output voltage signals of SO: $\mathbf{SO}_{\mathrm{R+G-}}$, $\mathbf{SO}_{\mathrm{G+R-}}$, $\mathbf{SO}_{\mathrm{Y+B-}}$, $\mathbf{SO}_{\mathrm{B+Y-}}$, $\mathbf{SO}_{\mathrm{Bri.}}$, $\mathbf{SO}_{\mathrm{Dar.}}$. 6 channels of SO voltage signal are input to a 6 × 3 cortex-processing module for matrix operation (Equation 5) to output the voltage signal of DO: $\mathbf{DO}_{\mathrm{R\&G}}$, $\mathbf{DO}_{\mathrm{B\&Y}}$, $\mathbf{DO}_{\mathrm{Gray}}$. Three channels of DO voltage signal are input to a 3 × 3 cortex-processing module for matrix operation (Equation 6) to output the processed RGB voltage signals.

Importance & significance: The color processing experiment seems to have two development prospects in computer vision/robot vision. Firstly, color constancy is one

of the amazing abilities of perceptual constancy of the human visual system (HVS), which enables the perceived color of objects largely constant as the light source color changes. In contrast, captured with regular digital cameras or videos, the physical color of scenes may be shifted by the varying external illuminant. One of the fundamental requirements in computer vision, especially for the robust color-based systems (e.g., color-based object recognition and tracking), is to extract reliable color cues that are invariant to the changes in external lighting[4]. A common solution is to first estimate the scene illuminant, which is then used to correct the color-biased images to get the so-called canonical images and also known as “white balance”. Traditional cameras only receive the intensity information of the three components of RGB to obtain the color information, and additional compensation algorithms are required for color constancy, which depend only on the statistical distributions of individual pixels and ignore their spatial contexts. However, replicating visual pathway of color vision in HVS, the hardware in our work could capture the strong dependencies between nearby pixels using the concentrically organized center-surround structure with both spectral and spatial opponency of the DO cells[3]. Therefore, the function of color constancy could be arranged in the hardware at the preprocessing period, which will simplify the complexity of subsequent processing circuits and algorithms. Secondly, for color-blindness patients, due to the weakening or obstruction of some color vision in related visual pathway, color cannot be accurately recognized, which will seriously affect the daily life. By understanding the causes of color blindness in the visual processing pathway at the hardware level, we can reverse design stimulus-related neural signals, and hopefully eliminate color blindness through brain-computer interface[5-6]. For the blind, artificial eyes that highly simulate visual pathways can reduce the additional circuit consumption of functions such as white balance, and can better integrate with the human visual system. Following this thought and designing hardware that is compatible with the human visual system, damaged neural structures can be replaced to stimulate the vision[7], which is similar to Elon Reeve Musk and Neuralink's next generation product “Blindsight”. It can be used to build electronic prosthetic eyes that match the visual system and help blind or color-blind patients regain normal color

vision.

Supplementary Note 3 | Principle and peripheral circuit design of Shape recognition hardware

3.1 Principle

Most of the receptive fields of the retina and LGN layers are round and respond most to the light spots in the center of the receptive field that match its size. Orientation selective neurons in V1 have receptive fields that extend along specific axes, with ON or OFF centers surrounded by antagonism on one or both sides. Simple cells in the cortex receive convergent input from the LGN with receptive fields aligned along a certain axis. The cortical neurons of V2/V4/IT extract the contour orientation information of local sites from simple cells, gradually converge to angles, and shapes, and make specific distinctions. Fragmented features generated in the retina are gradually integrated into recognizable forms in the visual cortex[8].

Abstracting the biological principle into a formula, the orientation convolution kernel (OCK) of a simple cell in V1 is defined as positive responsivity parallel to a specific axes and negative responsivity on both sides of the axes.

$$\mathbf{OCK}_{0^\circ} = \begin{bmatrix} - & - & - & - & - \\ - & - & - & - & - \\ + & + & + & + & + \\ - & - & - & - & - \\ - & - & - & - & - \end{bmatrix}$$

$$\mathbf{OCK}_{22.5^\circ} = \begin{bmatrix} - & - & - & - & - \\ + & - & - & - & - \\ - & + & + & + & - \\ - & - & - & - & + \\ - & - & - & - & - \end{bmatrix}$$

$$\mathbf{OCK}_{45^\circ} = \begin{bmatrix} + & - & - & - & - \\ - & + & - & - & - \\ - & - & + & - & - \\ - & - & - & + & - \\ - & - & - & - & + \end{bmatrix}$$

$$\mathbf{OCK}_{67.5^\circ} = \begin{bmatrix} - & + & - & - & - \\ - & - & + & - & - \\ - & - & + & - & - \\ - & - & + & - & - \\ - & - & - & + & - \end{bmatrix}$$

$$\mathbf{OCK}_{90^\circ} = \begin{bmatrix} - & - & + & - & - \\ - & - & + & - & - \\ - & - & + & - & - \\ - & - & + & - & - \\ - & - & + & - & - \end{bmatrix}$$

$$\mathbf{OCK}_{112.5^\circ} = \begin{bmatrix} - & - & - & + & - \\ - & - & + & - & - \\ - & - & + & - & - \\ - & - & + & - & - \\ - & + & - & - & - \end{bmatrix}$$

$$\mathbf{OCK}_{135^\circ} = \begin{bmatrix} - & - & - & - & + \\ - & - & - & + & - \\ - & - & + & - & - \\ - & + & - & - & - \\ + & - & - & - & - \end{bmatrix}$$

$$\mathbf{OCK}_{157.5^\circ} = \begin{bmatrix} - & - & - & - & - \\ - & - & - & - & + \\ - & + & + & + & - \\ + & - & - & - & - \\ - & - & - & - & - \end{bmatrix}$$

The OCKs in eight directions are constructed by giving "+" 0.1 A/W and "–" −0.02 A/W. The incident light of the regular hexagonal pattern is convolved with them to produce a photocurrent peak in the corresponding direction.

$$I = P_{\text{in}} * \mathbf{OCK} \tag{7}$$

The convergence of V1–V2–V4–IT is realized through a double-layer sparse neural network. The signal obtained by n-local convolution I_n is the input of the network.

The first layer of the sparse neural network is

$$\begin{bmatrix} H_1 \\ H_2 \\ H_3 \\ H_4 \\ H_5 \\ H_6 \\ H_7 \\ H_8 \end{bmatrix} = \begin{bmatrix} a_{11} & a_{12} & 0 & 0 & 0 \\ a_{21} & 0 & a_{23} & 0 & 0 \\ a_{31} & 0 & 0 & a_{34} & 0 \\ 0 & a_{42} & a_{43} & 0 & 0 \\ 0 & 0 & a_{53} & a_{54} & 0 \\ 0 & a_{62} & 0 & 0 & a_{65} \\ 0 & 0 & a_{73} & 0 & a_{75} \\ 0 & 0 & 0 & a_{84} & a_{85} \end{bmatrix} \begin{bmatrix} I_1 \\ I_2 \\ I_3 \\ I_4 \\ I_5 \end{bmatrix} \tag{8}$$

Here, a_{mn} represents the weight of the first layer at the mth row and n^{th} column, H_n is the output of the first layer.

Then activated by an activation function:

$$\begin{bmatrix} H_{1a} \\ H_{2a} \\ H_{3a} \\ H_{4a} \\ H_{5a} \\ H_{6a} \\ H_{7a} \\ H_{8a} \end{bmatrix} = \begin{bmatrix} a_1 & 0 & 0 & 0 & 0 & 0 & 0 & 0 \\ 0 & a_2 & 0 & 0 & 0 & 0 & 0 & 0 \\ 0 & 0 & a_3 & 0 & 0 & 0 & 0 & 0 \\ 0 & 0 & 0 & a_4 & 0 & 0 & 0 & 0 \\ 0 & 0 & 0 & 0 & a_5 & 0 & 0 & 0 \\ 0 & 0 & 0 & 0 & 0 & a_6 & 0 & 0 \\ 0 & 0 & 0 & 0 & 0 & 0 & a_7 & 0 \\ 0 & 0 & 0 & 0 & 0 & 0 & 0 & a_8 \end{bmatrix} \begin{bmatrix} H_1 \\ H_2 \\ H_3 \\ H_4 \\ H_5 \\ H_6 \\ H_7 \\ H_8 \end{bmatrix} \tag{9}$$

Here, a_n represents activation function for the nth output of the first layer, H_{na} is the nth output of the first layer after activation.

The second layer of the sparse neural network is:

$$\begin{bmatrix} O_1 \\ O_2 \\ O_3 \\ O_4 \end{bmatrix} = \begin{bmatrix} b_{11} & b_{12} & 0 & b_{14} & 0 & 0 & 0 & 0 \\ 0 & b_{22} & b_{23} & 0 & b_{25} & 0 & 0 & 0 \\ 0 & 0 & 0 & b_{34} & 0 & b_{36} & b_{37} & 0 \\ 0 & 0 & 0 & 0 & b_{45} & 0 & b_{47} & b_{48} \end{bmatrix} \begin{bmatrix} H_{1a} \\ H_{2a} \\ H_{3a} \\ H_{4a} \\ H_{5a} \\ H_{6a} \\ H_{7a} \\ H_{8a} \end{bmatrix} \tag{10}$$

Here, b_{mn} represents the weight of the second layer at the mth row and nth column, O_n is the output of the second layer.

Based on the sparse neural network, the algorithm for shape recognition is constructed. First, we create a database (Fig. S15a). Gaussian noise is added to the right triangles, to form a database (1600 groups in total, 400 groups for each) according to the labels $[T_1 \quad T_2 \quad T_3 \quad T_4]^T$. The right triangle pattern is divided into five regions (Fig. 3c), which are convolved with a specific OCK respectively (Equation 7) to obtain the input data I_n of the sparse neural network. The dataset is divided into 7:3 ratios for training and validation, respectively. For each training, the input data I_n performs the first-layer matrix multiplication (Equation 8), activation (Equation 9), and second-layer matrix multiplication (Equation 10) to obtain the output $[O_1 \quad O_2 \quad O_3 \quad O_4]^T$. Comparing outputs with the labels $[T_1 \quad T_2 \quad T_3 \quad T_4]^T$, the cross-entropy loss is calculated, and the weights of each level are updated by the gradient descent method. The recognition rate is calculated by counting the proportion of feedforward calculation results that are the same as the label in the validation dataset. Furthermore, the selection of activation functions between the first and second levels has a certain impact on loss

function and recognition accuracy. In our study, we used 9 activation functions, including ELU, ReLU, CELU, SELU, PReLU, Mish, Hardswish, Sigmoid, and Tanh, to train the fully and sparsely connected neural network. The recognition rate and loss after 30 epochs of training were counted (supplementary Figs. S15d, e). Considering the recognition rate and the feasibility of hardware implementation of the activation function, we chose Mish as the activation function of the sparse neural network (Fig. S15f).

3.2 Circuit design

The shape recognition hardware (Fig. S14) uses two modules that are the same as the color processing hardware and requires one retina-sensing module (reused five times at 5 × 5) and three cortex-processing modules (one each for 5 × 8, 8 × 8, and 8 × 4). Among them, the retina-sensing module is modulated into five specific OCKs through gate voltage pulse, and convolving with the corresponding area of the triangle light input to produce five voltage signals as inputs of the neural network (Equation 7). Limited by small-light-spot input of experimental setup, the retina-sensing module is reused to record five voltage signals which are imported into subsequent circuits through a signal generator in experiment. The signals are connected to the 5 × 8 cortex-processing module for matrix operation (Equation 8). The eight-channel output voltage signals are input to the 8 × 8 cortex-processing module for activation (Equation 9). The eight-channel output voltage signals are input to the 8 × 4 cortex-processing module for matrix operation (Equation 10) to output four-channel voltage signals. According to the comparison of the channel number of the maximum value with the label, the loss and recognition rate are calculated in experiment to update the weights and perform the next epoch until they get convergence. The Gaussian blur-like illumination input generated by the light mask consisting of a right-angle triangle mask and the frosted glass is used for the actual experimental measurement. The recognition rate of each epoch in experiment (Fig. 3f) is obtained by counting the results under 480 (30% of 1,600) different Gaussian blur-like illumination inputs by moving the glass.

Supplementary Note 4 | Principle and peripheral circuit design of Motion tracking

hardware

4.1 Principle

The HVS's perception and judgment of motion information come from various regions of the visual pathway. In the retina and visual cortex, some neurons are sensitive to the direction of movement. They both receive signals from neurons arranged in a certain direction at the previous level with different transmission time due to different axon lengths. When the movement direction of the light stimulus is consistent with the direction of axon shortening, the integrated multi-channel signals from the previous stage will superimpose, whose intensity can exceed the threshold to enable activation, and the signal can be subsequently transmitted. Otherwise, the threshold cannot be exceeded and the signal is no longer transmitted, realizing the perception and judgment of the specific direction of movement. The core of CSRF is that the post-stage pathway is the integration of multi-channel signals from the pre-stage. For the direction selector, the direction-related delay is added to each signal of the pre-stage. It mainly functions in the visual cortex with a larger receptive field.

In the visual cortex, beyond the middle temporal (MT) cortex with movement sensitivity, the medial superior temporal (MST) cortex can direct eye movements according to information from MT for object tracking[9].

4.2 Circuit Design

We designed the circuits and fabricated the hardware for the direction selector and the 1D bidirectional motion tracker (Fig. S16).

Direction Selector: All drains of the retina-sensing module are connected to the Ground, and each row of sources is connected separately, working with the photovoltaic effect. The four-channel signals of the source are connected to the TIAs, which converts the photovoltaic current into voltage. The delay modules ($\Delta t_{1\text{–}4}$, the long-delay circuit constructed by the 555 timer) are added to each channel respectively. The output signal is judged through a four-input AND gate. According to the relative value of $\Delta t_{1\text{–}4}$, the direction selector for the specific direction is obtained. In experiment, $\Delta t_{1\text{–}4}$ is set to 100 μs/25 ms/50 ms/75 ms by adjusting the resistor at 100 Ω/25 kΩ/50 kΩ/75 kΩ and a 1 μF capacitor, respectively, in the delay module.

1D Bidirectional Motion Tracker: Each drain of the 10 × 10 retina-sensing module is connected to the Ground, and each row of sources is separately connected to a TIA to form ten-channel photovoltaic voltage outputs. The ten-channel outputs are connected to the drain of the switch transistor array (10 × 3), whose sources are connected to the voltage dividing resistor to obtain three-channel voltage outputs. The middle one is connected to the delay module, whose output signal is connected to the dual-input AND with the signals on both sides respectively to obtain the movement direction signal V_{sf}. The V_{sf} of up and down movement direction is connected to the left and right shift signal control terminal of the bidirectional shift register respectively to control outputs of the register, which modulates the gate and on/off status of the switch transistor array. The gating of the switch transistor depends on the movement direction of the light stimulus received by the retina-sensing module. The output terminal of the shift register records the position of the light stimulus for real-time motion tracking and location.

Supplementary Figures

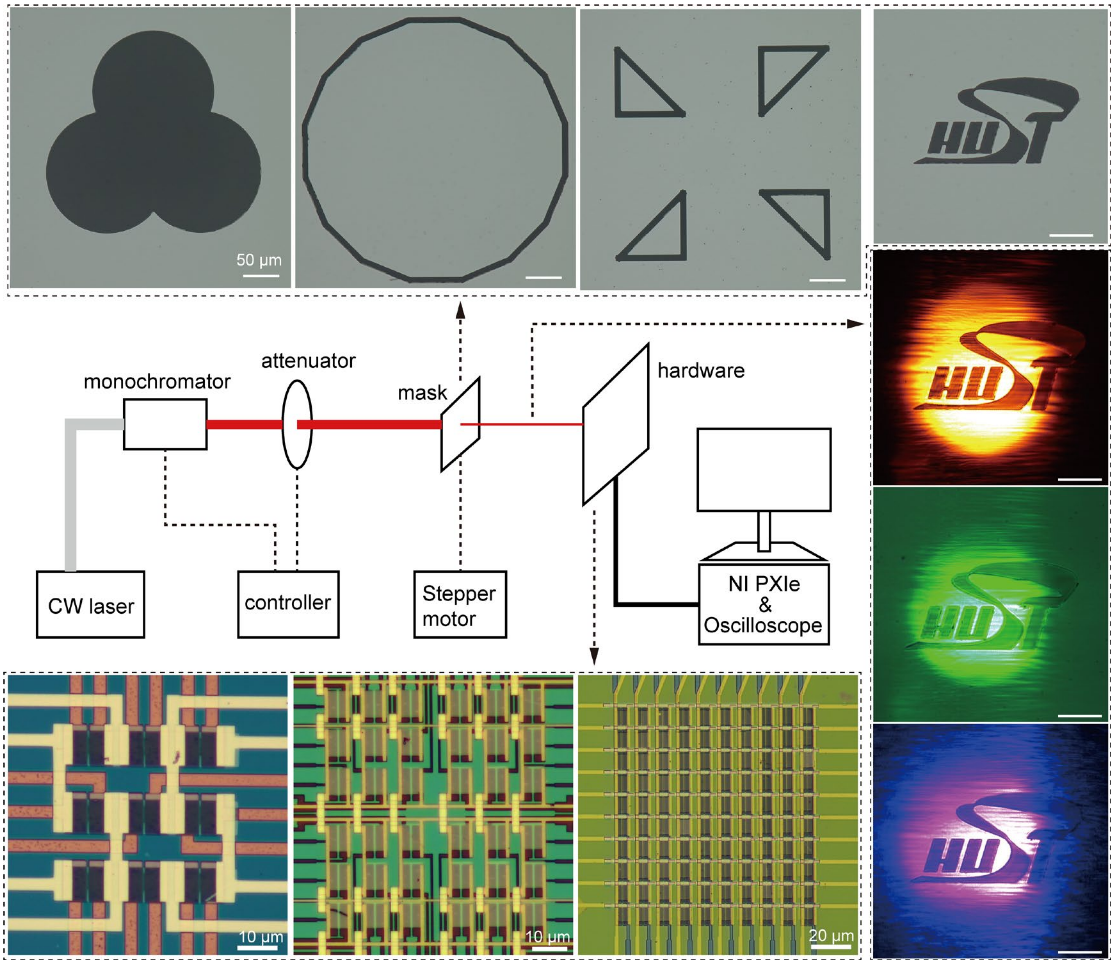

Fig. S1 | Schematic diagram of the experimental setup. Top: microscope image of the light mask. Scale bar, 50 μm. The left one is used for color processing. The regular hexadecagon of 50 × 50 pixels (middle) and four 15 × 15–pixel size right-angled triangle (right) ones are used for shape recognition. Bottom: microscope image of the SFG array. Scale bar, 10/10/20 μm. Right: microscope image of the light mask with RGB light (the spot diameter is about 150 μm) under the experiment for motion tracking, where the pattern "hust" is transparent and other places are opaque. Scale bar, 50 μm.

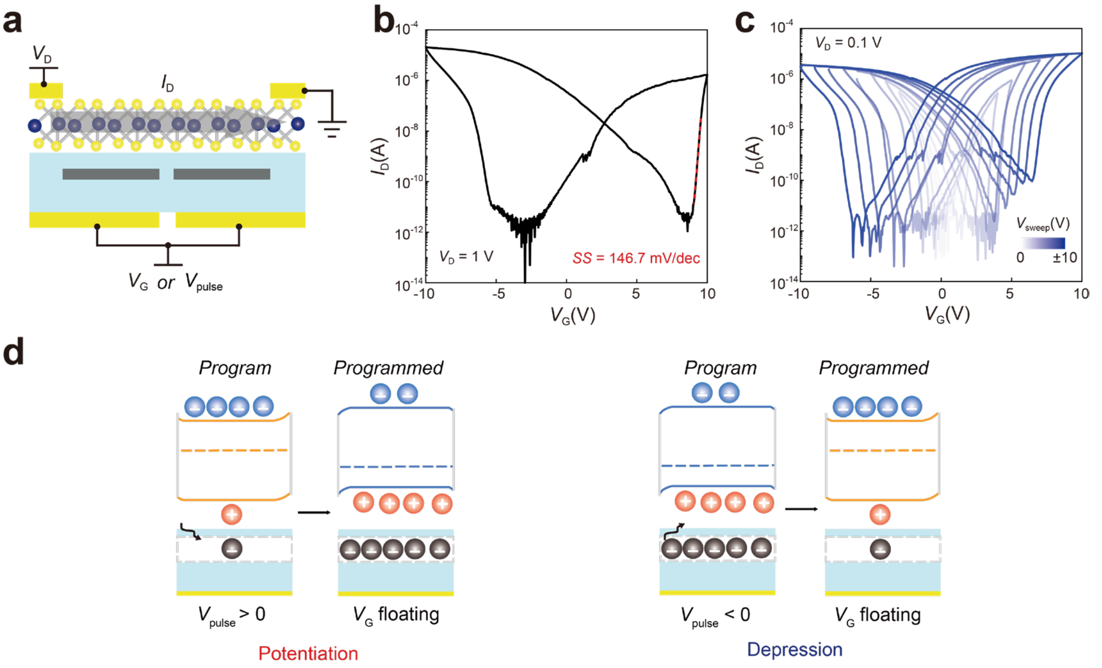

Fig. S2 | Example of reconfigurable floating gate modulation. a, Schematic diagram of the experimental setup, $V_{G1} = V_{G2} = V_G$. **b,** Transfer curve (V_D = 1 V). **c,** Transfer curves (V_D = 0.1 V) under different sweep gate voltage ranges. **d,** Schematic diagram of the band structure of floating gate modulation: electrons in the floating layer are increased by the positive gate V_{pulse}, which is equivalent to electrostatic modulation by the negative gate voltage, causing more holes programmed in the channel with P-type conductivity. N-type conductivity is obtained by the negative gate V_{pulse} on the contrary.

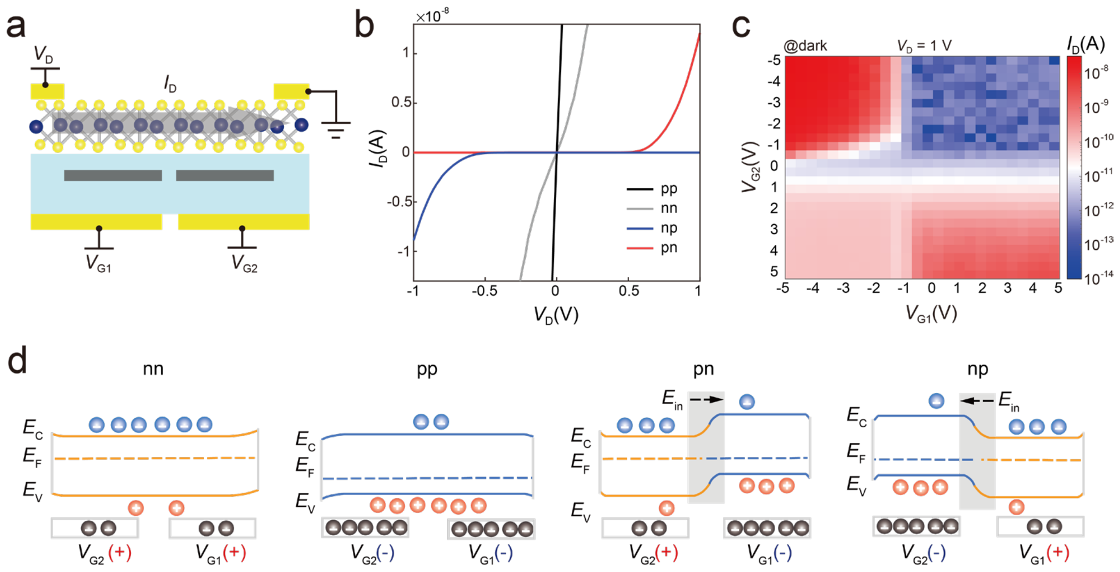

Fig. S3 | Example of reconfigurable p–n junction. a, Schematic diagram of experimental setup, dual gate voltages are controlled independently. **b,** Output curves of various modulation states (p–p/n–n/n–p/p–n): negative (positive) V_{pulse}, which is equivalent to positive (5 V) or negative (−5 V) V_G, modulates N (P)-type conduction. **c,** I_D–V_{G1} & V_{G2} under dark state. (equivalent gate voltage, V_D = 1 V). **d,** Schematic diagram of the band structure corresponding to four types of p–n junction in **b**. The symbol of $V_{G1(2)}$ is the equivalent gate voltage. The positive gate voltage induces the floating gate to provide fewer electrons that produce fewer channel mirror holes, and the channel is modulated to N-type with the dominant electrons. The negative gate voltage induces the floating gate to provide more electrons that produce more channel mirror holes, and the channel is modulated to P-type with the dominant holes. Between the locally modulated N-type and P-type channels, the carrier diffusion under the concentration difference and drift under the built-in electric field are balanced to form the p-n junction.

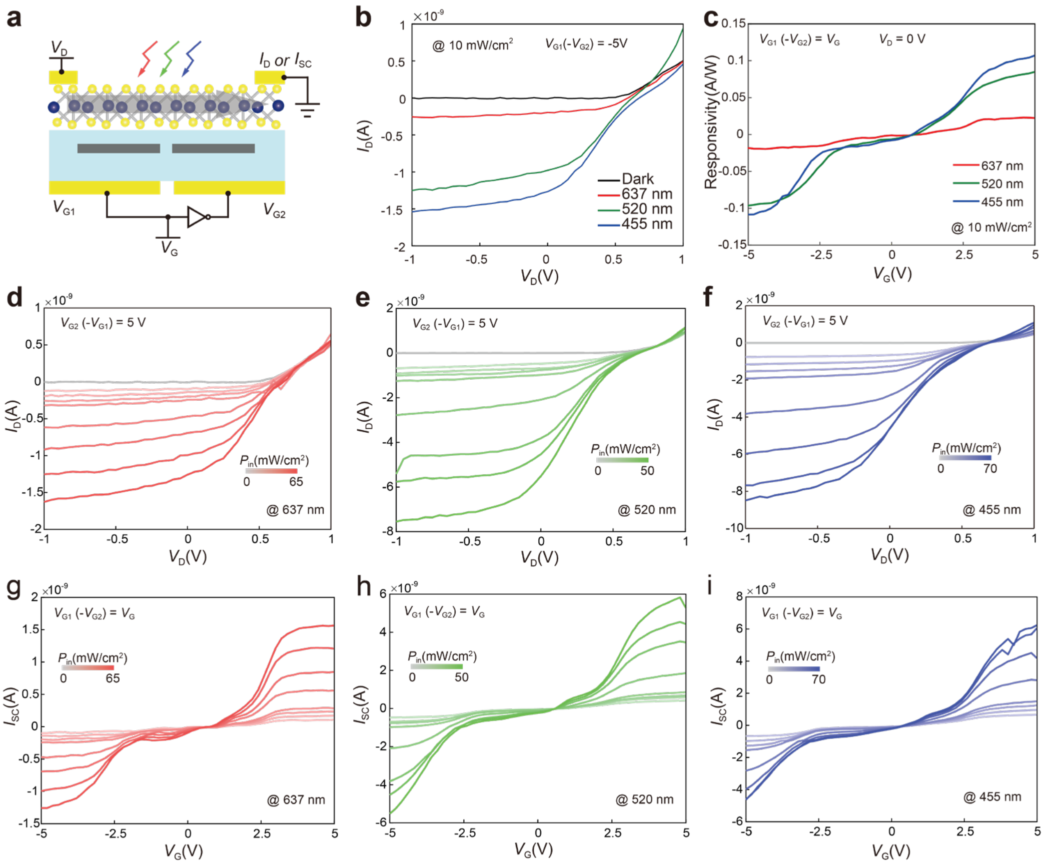

Fig. S4 | Example of optoelectronic characteristics for reconfigurable p–n junction. a, Schematic diagram of experimental setup, $V_{G1} = -V_{G2} = V_G$. **b,** I_D–V_D curves under red (637 nm) / green (520 nm) / blue (455 nm) light (P_{in} = 10 mW/cm^2, $V_G = -5$ V) and dark, which are extracted from output curves of photovoltaic effect under the light of different P_{in} and wavelength 637 nm(**d**)/520 nm(**e**)/455 nm(**f**). **c,** Optical responsivity with the equivalent gate voltage (P_{in} = 10 mW/cm^2, V_D = 0 V), which is extracted from I_{SC}–V_G under the light of different P_{in} and wavelength 637 nm(**g**)/520 nm(**h**)/455 nm(**i**).

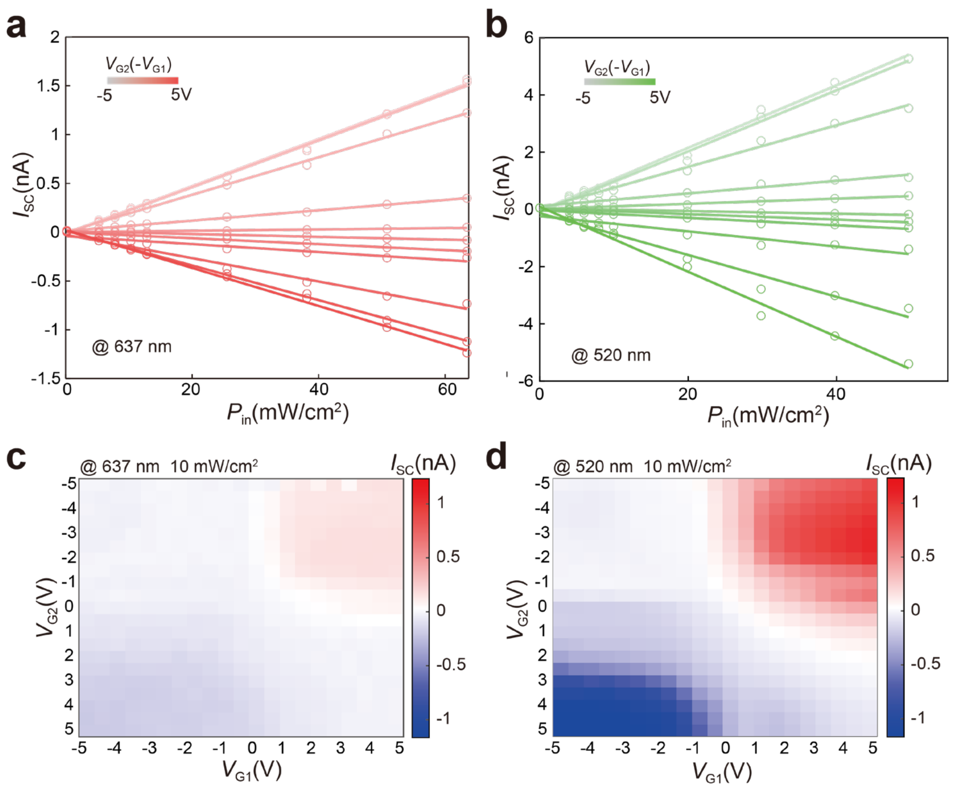

Fig. S5 | Example of optoelectronic characteristics for p–n junction (continued). a–b, Dependence of the short-circuit photocurrent on the light intensity for different split-gate equivalent voltages under the light of 637 (**a**)/520 nm (**b**). **c–d,** Dependence of the short-circuit photocurrent on the split-gate equivalent voltages under the light of 637 (**c**)/520 nm (**d**) and 100 W/m^2.

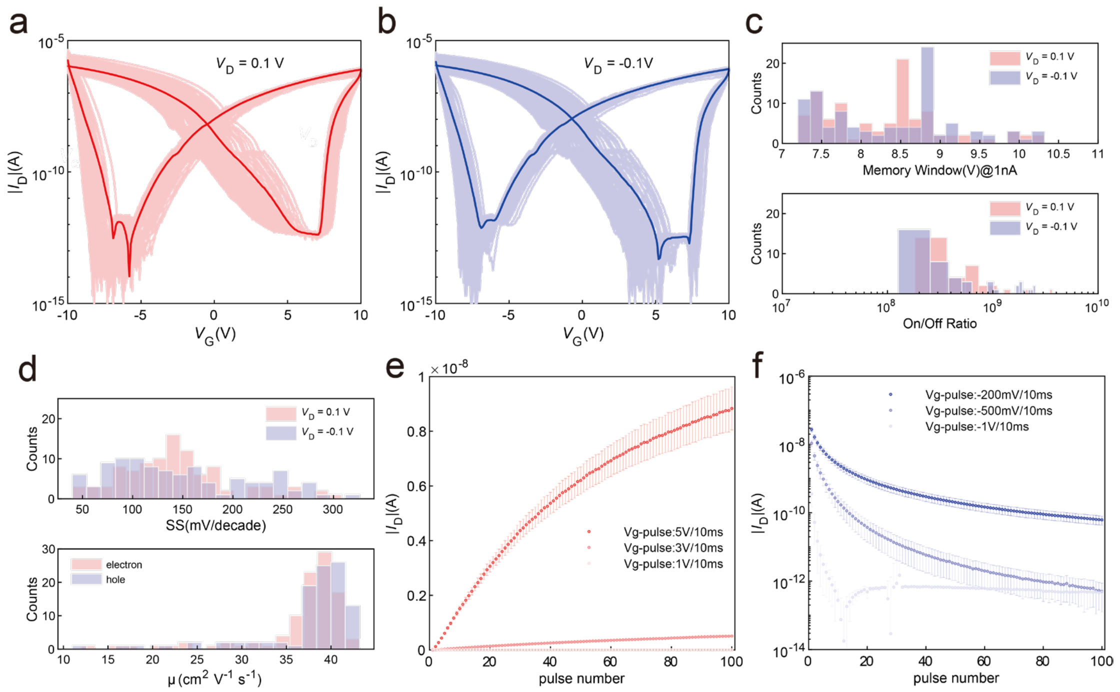

Fig. S6 | Statistical results for floating-gate transistor characteristics of unit devices. a–b, Transfer curves of 100 devices at ± 10V sweep gate voltage ($V_{G1} = -V_{G2} = V_G$, V_D = 0.1 V / −0.1 V, respectively). **c.** Histogram of the memory window @ I_D = 1 nA (top) and the transistor ON/OFF ratio (bottom). **d,** Histogram of the subthreshold swing (SS) (top) and WSe_2 carrier mobility (bottom). **e–f,** Drain currents modulated by 100 gate voltage V_{pulse} of 5/2/1/−0.2/−0.5/−1V and 10 ms, error bar is extracted from 100 units of the 10 × 10 array. The error bars represent the standard deviation.

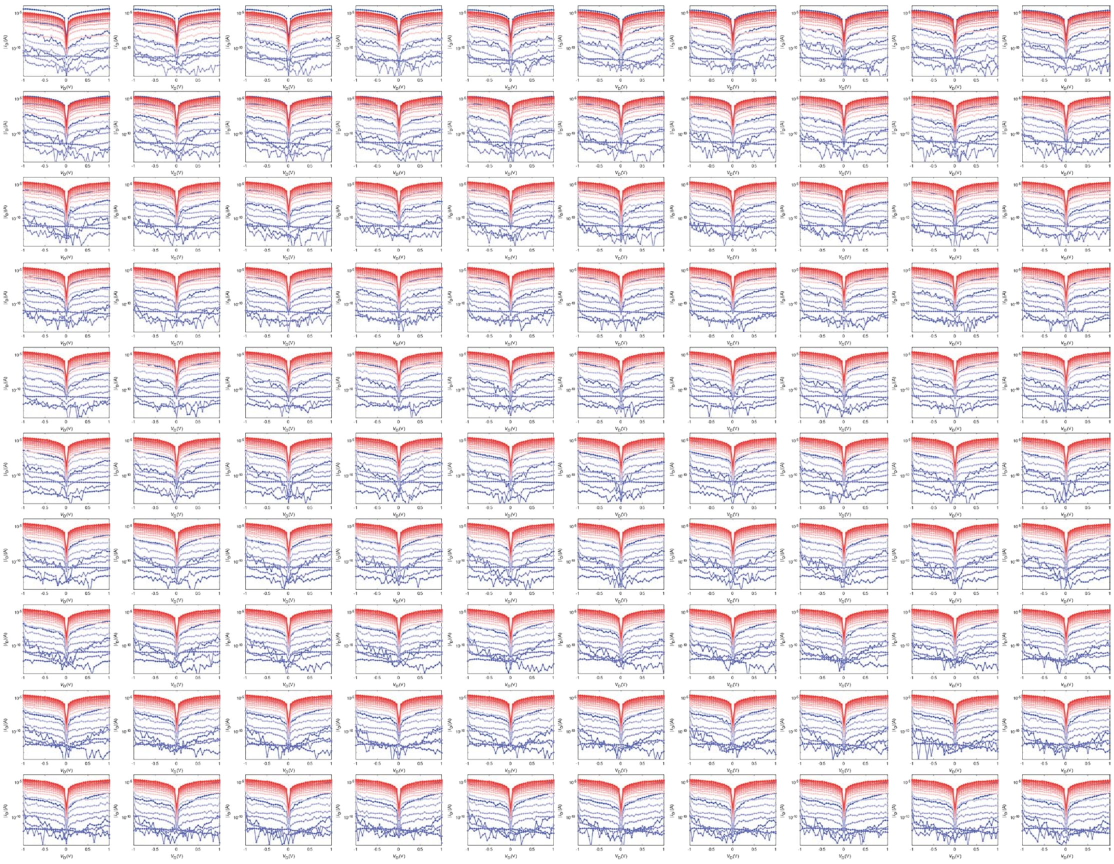

Fig. S7 | Output curves of 100 units. $V_{G1} = -V_{G2} = V_G$, changing from −10 V to 10 V with a step of 1 V.

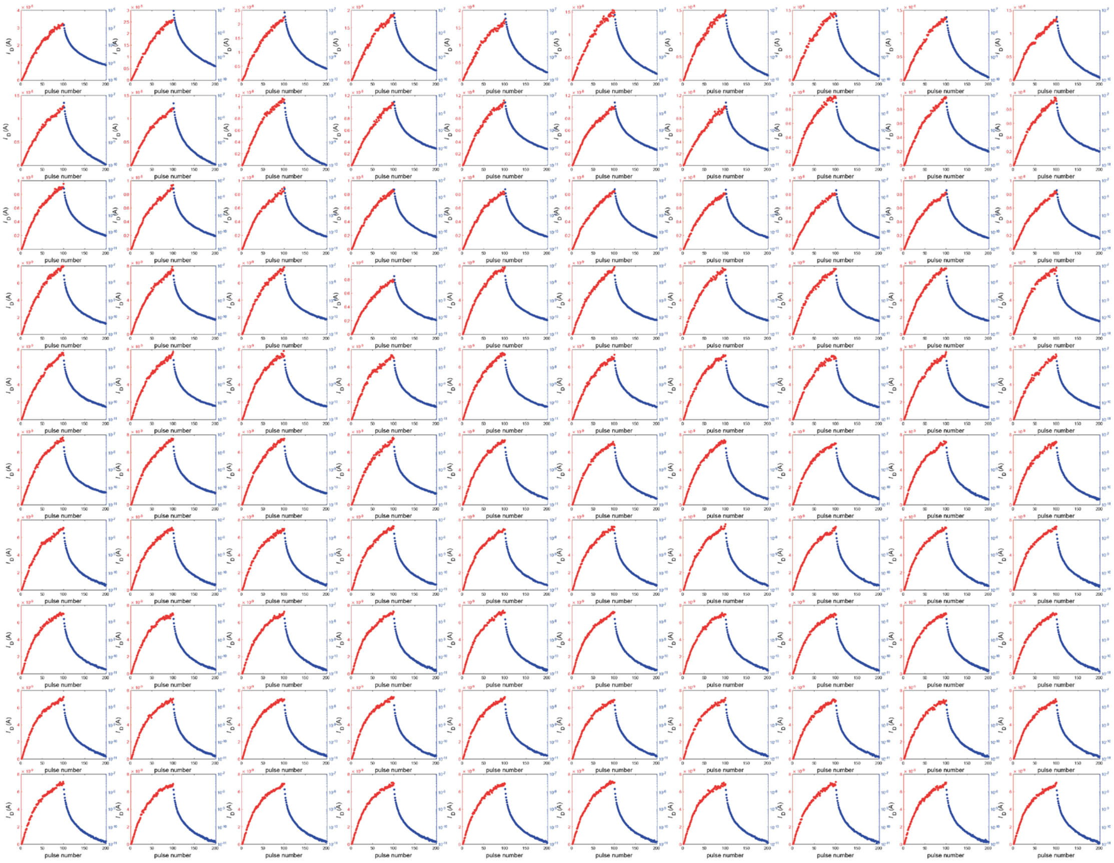

Fig. S8 | Nonvolatile and programmable conductivity under a series of gate voltage pulses for 100 units. The first (last) 50 V_{pulse} are 5 V/10 ms (−0.2 V/10 ms).

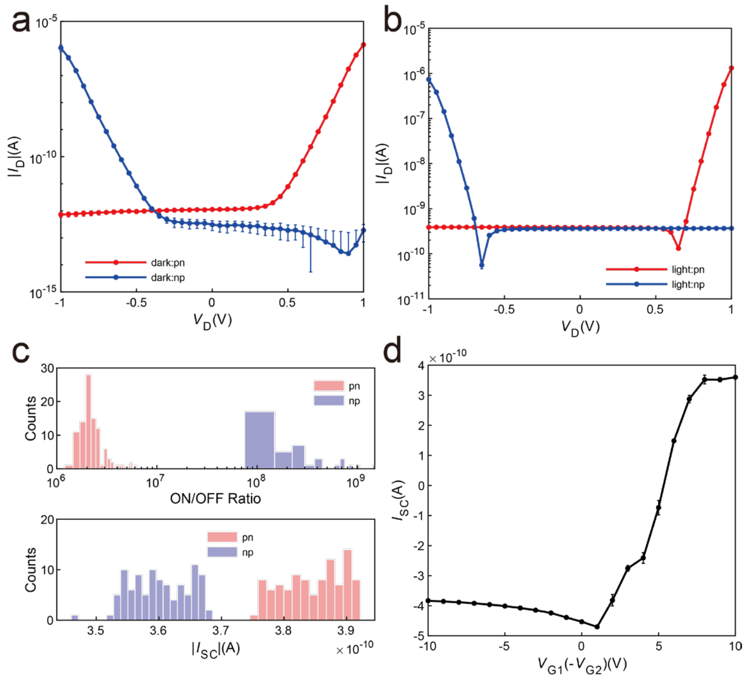

Fig. S9 | Statistical results for p–n junction characteristics of unit devices. a–b, Output curves of p-n/n-p configurations (equivalent gate voltages $V_{G1} = -V_{G2} = \pm 10$ V) from 100 units in the dark (**a**) and under optical illumination (**b**, CCD light source). **c,** Distributions of the ON/OFF ratio (top) and the short-circuit photocurrents (bottom). **d,** Dependence of the short-circuit photocurrent on the split-gate equivalent voltages ($V_{G1} = -V_{G2}$). The error bars in **a** and **d** represent the standard deviation.

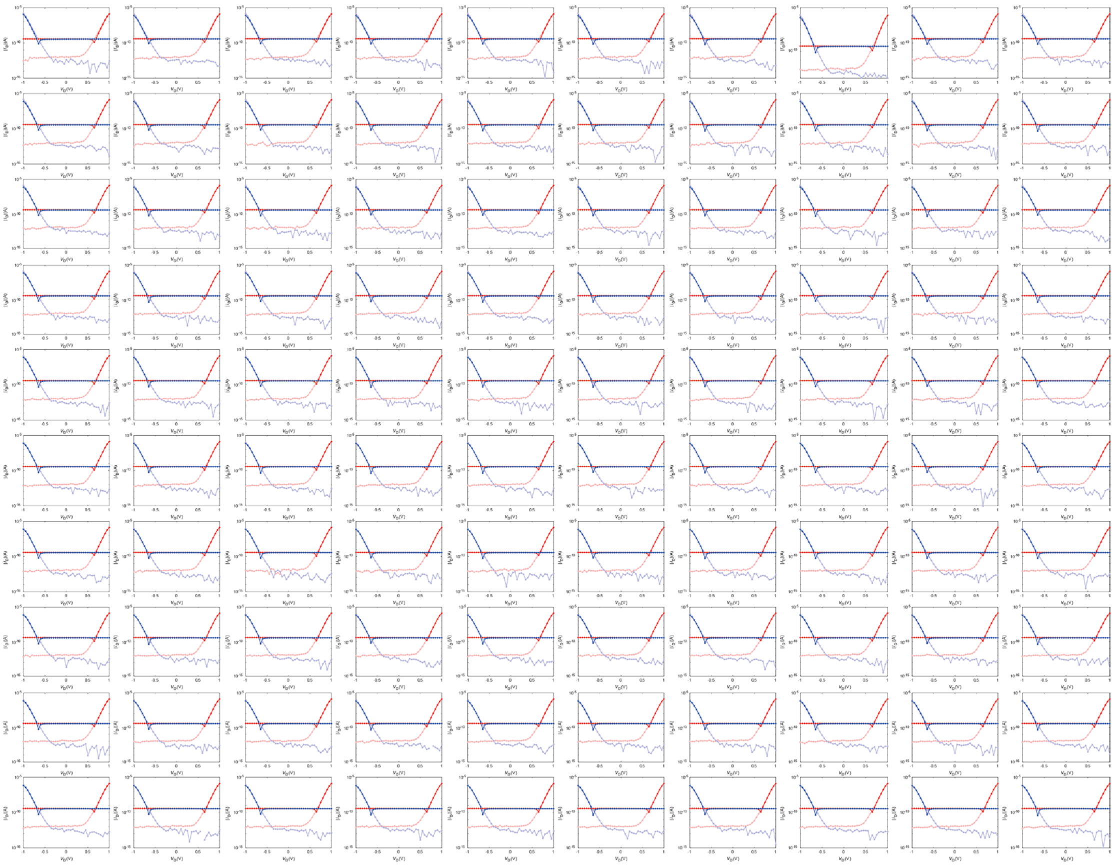

Fig. S10 | Output curves of p–n/n–p configurations under dark and optical illumination for 100 units. Equivalent gate voltages $V_{G1} = -V_{G2} = -10$ V (10 V) for p–n (n–p) junction.

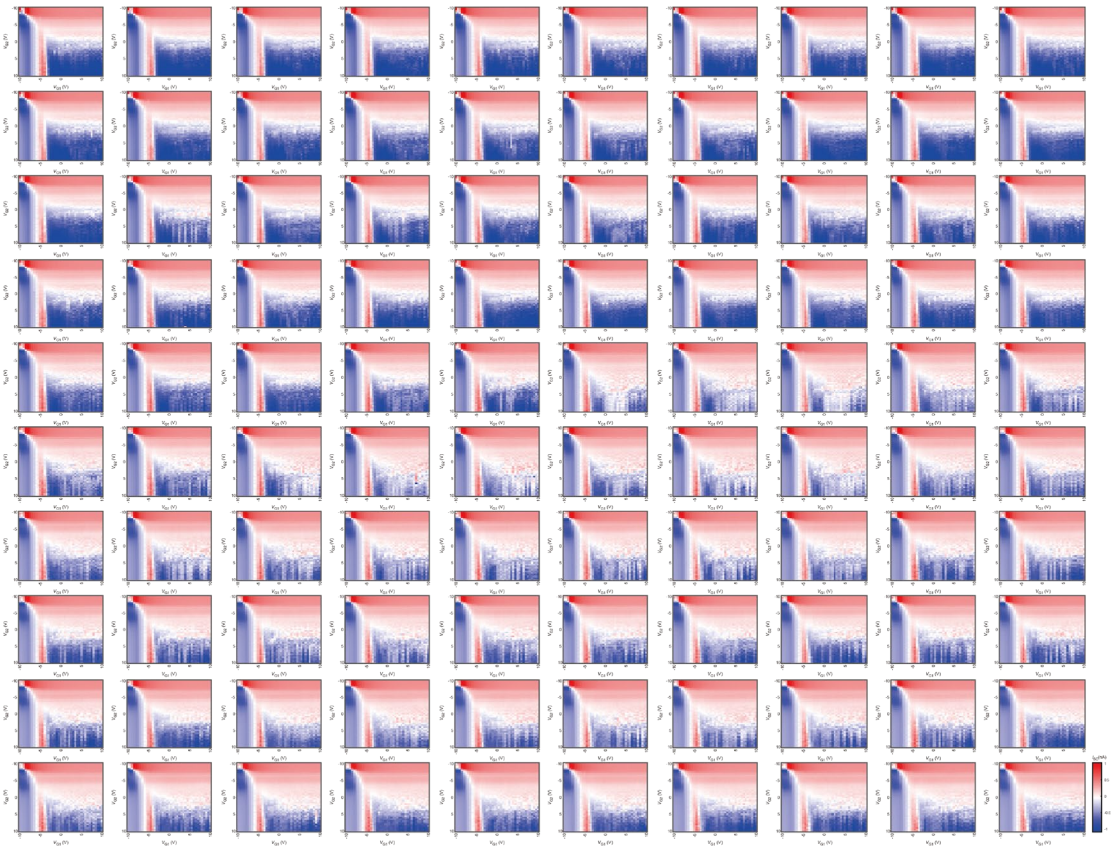

Fig. S11 | Dependence of the short-circuit photocurrent on the split-gate equivalent voltages for 100 units. Equivalent gate voltages V_{G1} and V_{G2} range from −10 to 10 V with a step of 0.05 V. The array is illuminated by the white light source.

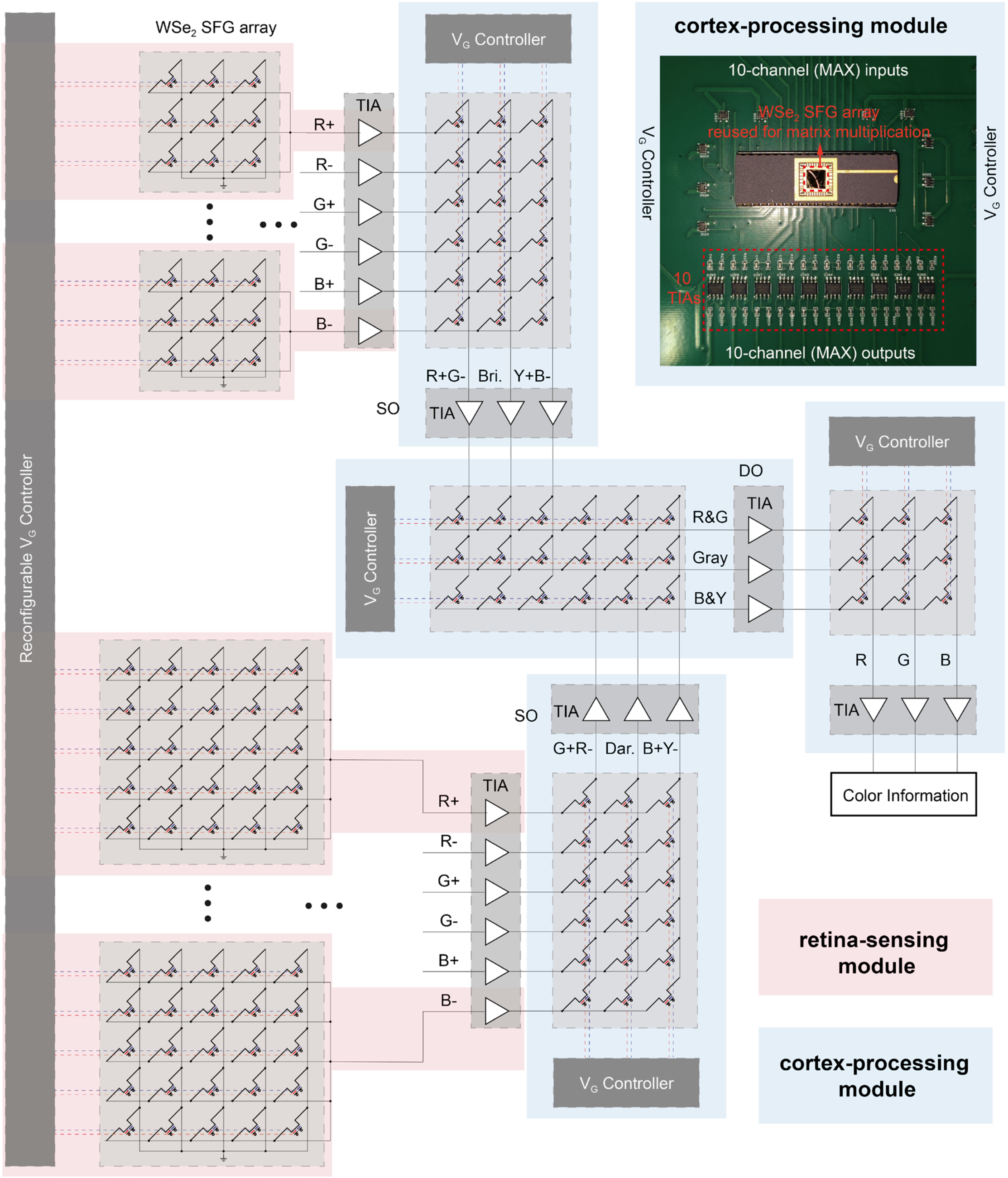

Fig. S12 | Circuit diagram of color processing hardware. retina-sensing module: consists of a 10 × 10 SFG array and a TIA. Each column is connected to the ground, and each row is connected to the TIA to convert to voltage. Cortex-processing module: consists of a 10 × 10 SFG array and 10 TIAs. Each column is connected to input from the pre-stage, and each row is connected to a TIA to perform matrix multiplication. The macroscopic image: cortex-processing module.

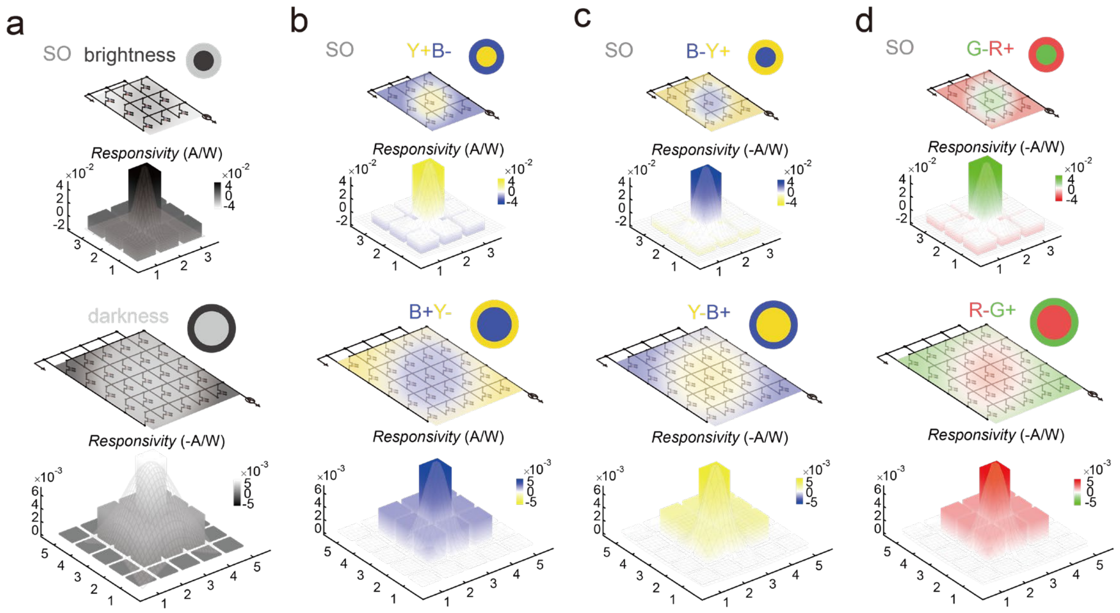

Fig. S13 | Hardware photoresponsivity distributions corresponding to SO CSRF. For black–white (**a**), yellow–blue (**b**), blue–yellow (**c**), and green–red (**d**) (top: small CSRF; bottom: large CSRF).

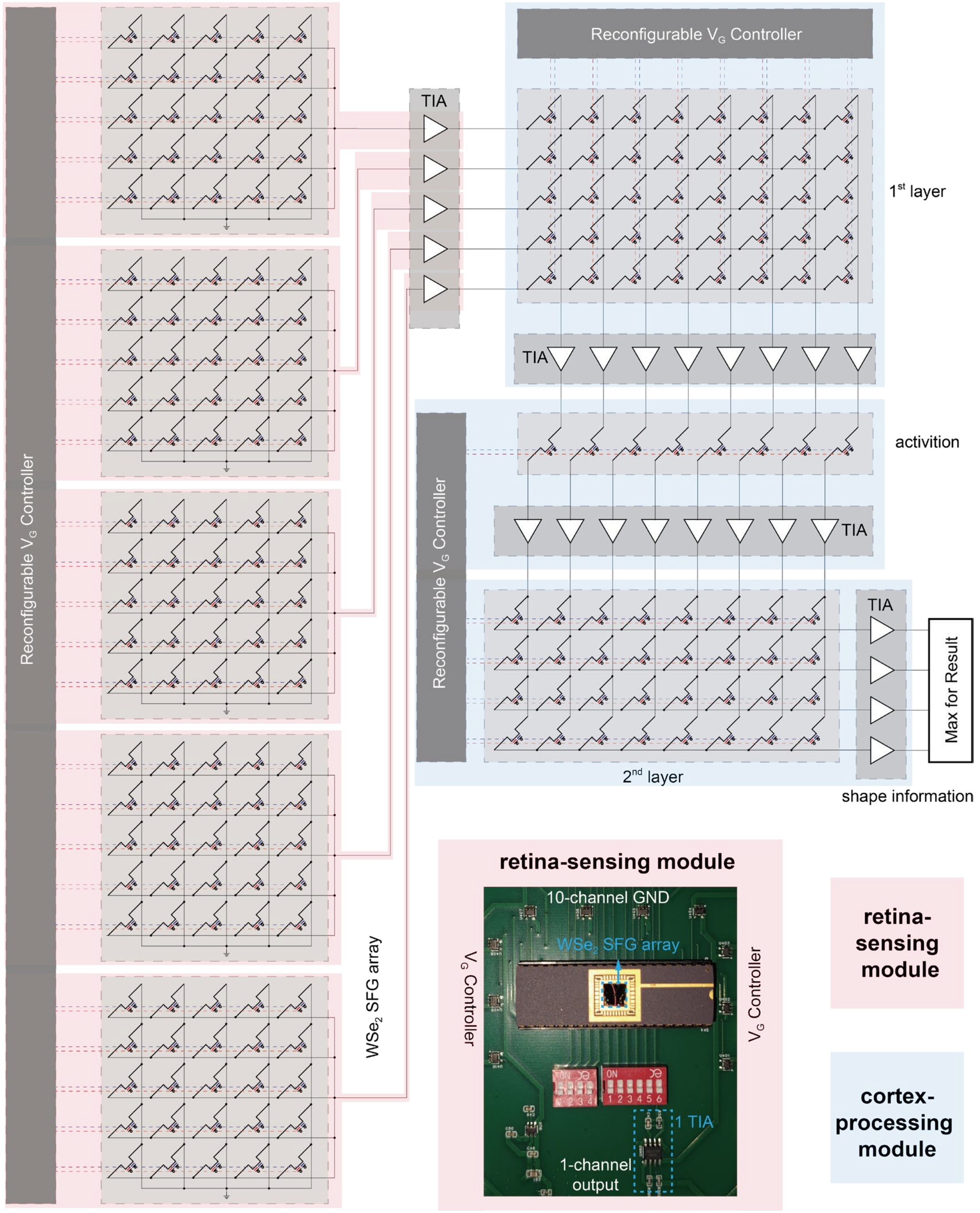

Fig. S14 | Circuit diagram of shape recognition hardware. The macroscopic image: retina-sensing module.

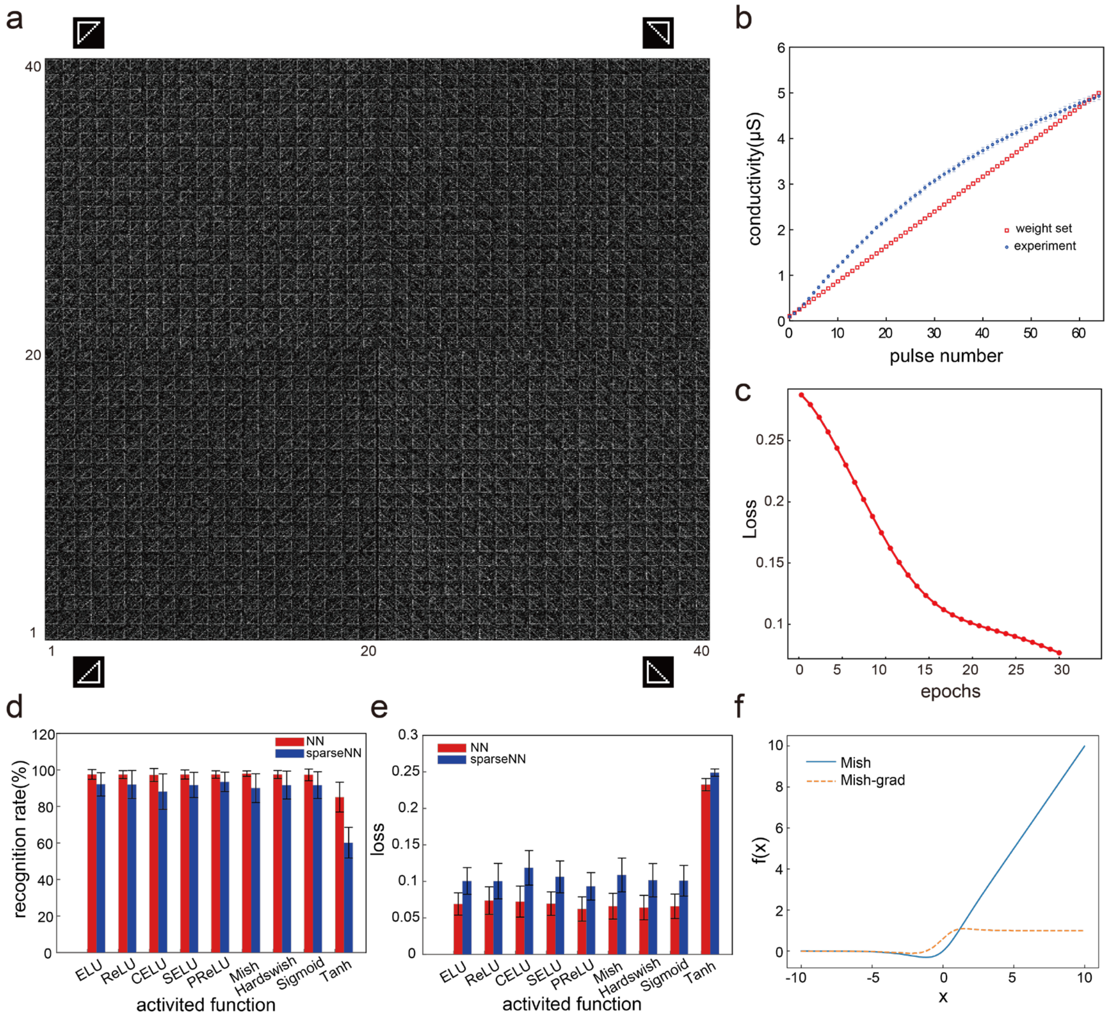

Fig. S15 | Results related to shape recognition algorithms. a, Datasets for training with a Gaussian noise level of 0.8. **b,** The 64-level conductivity weights of the hardware for the sparse neural network under the condition of setup (red mark) and experiment (blue mark). **c,** Loss during 30 epochs of training. **d–e,** Recognition rate and loss of the 30th epoch by fully connected neural network and sparse neural network simulated using different activation functions. The error bars represent the standard deviation. **f,** Activation function Mish.

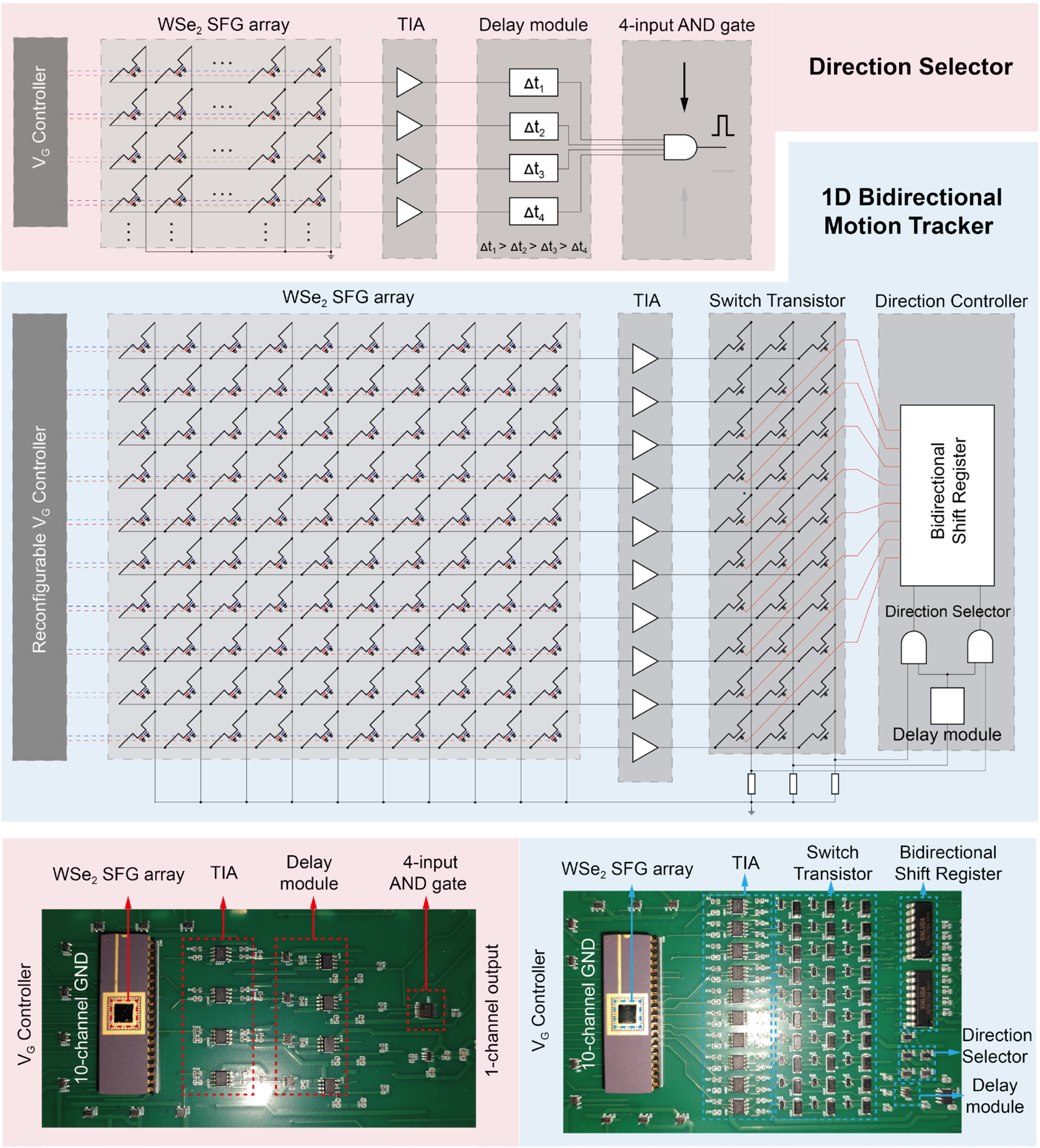

Fig. S16 | Circuit diagram of motion tracking hardware. Direction Selector (top), 1D Bidirectional Motion Tracker (middle), and their macroscopic image (bottom).

Supplementary References

1. Nathans, J. The evolution and physiology of human color vision: Insights from molecular genetic studies of visual pigments. *Neuron* **24**, 299–312 (1999).
2. Gegenfurtner, K. R. Cortical mechanisms of colour vision. *Nat. Rev. Neurosci.* **4**, 563–572 (2003).
3. Gao, S. B., Yang, K. F., Li, C. Y. & Li, Y. J. Color Constancy Using Double-Opponency. *IEEE Trans. Pattern Anal. Mach. Intell.* **37**, 1973–1985 (2015).
4. Gijsenij, A., Gevers, T. & Van De Weijer, J. Computational color constancy: Survey and experiments. *IEEE Trans. Image Process.* **20**, 2475–2489 (2011).
5. Luo, X. et al. A bionic self-driven retinomorphic eye with ionogel photosynaptic retina. *Nat. Commun.* **15**, 1–9 (2024).
6. Yang, R. et al. Assessment of visual function in blind mice and monkeys with subretinally implanted nanowire arrays as artificial photoreceptors. *Nat. Biomed. Eng.* 1–22 (2023) doi:10.1038/s41551-023-01137-8.
7. Beauchamp, M. S. et al. Dynamic Stimulation of Visual Cortex Produces Form Vision in Sighted and Blind Humans. *Cell* **181**, 774–783 (2020).
8. Hubel, D. H. & Wiesel, T. N. Receptive fields and functional architecture of monkey striate cortex. *J. Physiol.* **195**, 215–243 (1968).
9. Priebe, N. J. & Ferster, D. Inhibition, Spike Threshold, and Stimulus Selectivity in Primary Visual Cortex. *Neuron* **57**, 482–497 (2008).
