# Supplementary material for: Multifunctional human visual pathway-replicated hardware based on 2D materials
Source: Nat Commun. 2024 Oct 5;15:8650. doi: 10.1038/s41467-024-52982-3 (PMC11455896; doi:10.1038/s41467-024-52982-3)
Supplement: Supplementary file 2 — Description of Additional Supplementary Information [file 41467_2024_52982_MOESM2_ESM.docx]

**Description of Additional Supplementary Files**

File Name: Supplementary Movie 1

Description: Demonstration of the composition and experimental setup of optoelectronic measurement system with a recorded measurement video.
